# Photoluminescence of monovalent indium centres in phosphate glass

**DOI:** 10.1038/srep13646

**Published:** 2015-09-01

**Authors:** Hirokazu Masai, Yasuhiro Yamada, Shun Okumura, Takayuki Yanagida, Yutaka Fujimoto, Yoshihiko Kanemitsu, Toshiaki Ina

**Affiliations:** 1 Institute for Chemical Research, Kyoto University, Gokasho, Uji, Kyoto 611-0011, Japan; 2 Nara Institute of Science and Technology, 8916-5, Takayama-cho, Ikoma, Nara, 630-0192 Japan; 3 Department of Applied Chemistry, Tohoku University, 6-6-07 Aoba, Sendai 980-8579, Japan; 4 Japan Synchrotron Radiation Research Institute (JASRI/SPring-8), Kouto, Sayo-cho, Hyogo 679-5198, Japan

## Abstract

Valence control of polyvalent cations is important for functionalization of various kinds of materials. Indium oxides have been used in various applications, such as indium tin oxide in transparent electrical conduction films. However, although metastable In^+^ (5 s^2^ configuration) species exhibit photoluminescence (PL), they have attracted little attention. Valence control of In^+^ cations in these materials will be important for further functionalization. Here, we describe In^+^ species using PL and X-ray absorption fine structure (XAFS) analysis. Three absorption bands in the UV region are attributed to the In^+^ centre: two weak forbidden bands (^1^*S*_0_ → ^3^*P*_1,_^1^*S*_0_ → ^3^*P*_2_) and a strong allowed band (^1^*S*_0_ → ^1^*P*_1_). The strongest PL excitation band cannot be attributed to the conventional allowed transition to the singlet excited state. Emission decay of the order of microseconds suggests that radiative relaxation occurs from the triplet excitation state. The XAFS analysis suggests that these In^+^ species have shorter In–O distances with lower coordination numbers than in In_2_O_3_. These results clearly demonstrate that In^+^ exists in a metastable amorphous network, which is the origin of the observed luminescent properties.

Indium oxide is an important metal oxide that has been used in various applications such as transparent electrical conduction films^1^,^2^,^3^,^4^,^5^, ferromagnetic devices^6^,^7^, and sensing^8^,^9^. Among these functional materials, transparent electrical conduction films, which accounts for global consumption of indium despite its rarity, are essential in our daily life. However, it is assumed that all In species in such materials are in the trivalent state. Therefore, the metastable monovalent In species (In^+^) has not been recognised as a component in oxide materials.

On the other hand, In^+^ is used as an *n*s^2^-type centre in halide phosphors. The *n*s^2^-type ions (*n* = 4, 5, 6) are light-emitting ions exhibiting an *n*s^2^ electron configuration in the ground state and an *n*s^1^*n*p^1^ configuration in the excited state^10^. The emissions properties of several *n*s^2^-type ions have been reported, including alkali halides^11^,^12^,^13^,^14^,^15^,^16^,^17^,^18^,^19^,^20^,^21^,^22^,^23^,^24^,^25^ and several oxides^26^,^27^,^28^,^29^,^30^,^31^,^32^,^33^,^34^,^35^,^36^,^37^,^38^,^39^,^40^,^41^,^42^,^43^,^44^,^45^,^46^,^47^. In contrast to conventional 5 s^2^ centres such as Sb^3+^ and Sn^2+^ for which the emissions properties of In oxides have been reported^31^,^32^,^33^,^34^,^35^,^36^,^37^,^38^,^39^,^40^,^41^,^42^,^43^,^44^,^45^,^46^,^47^, emissions have not been studied for the In^+^ centre in powdered oxide phosphor. Although there is one report on the photoluminescence (PL) of In^+^ in oxide crystals^48^, the decay constant is quite different from that observed for conventional *n*s^2^-type centres due to spin-forbidden relaxation (^3^*P*_1_ → ^1^*S*_0_). Moreover, emissions of In^+^ in oxide glass have not been studied.

From the standpoint of thermodynamic stability, it is possible that PL properties could arise from emissions centres in a metastable host matrix, *i.e.* an oxide glass with a random network. Design of phosphor materials has been mainly limited to rare earth-containing powdered crystals, because the coordination state can be easily controlled compared with other emissions centres. Considering both the site distribution of amorphous glass and the confinement effects of the surrounding amorphous region, such metastable centres could exhibit PL in amorphous glasses, although this has not been demonstrated using conventional crystalline phosphor powders.

Recently, we have studied the PL of *n*s^2^-type (*n* = 5) centres such as Sn^2+^ 39,40,41,42,43,44,45, Sb^3+^ 46, and Te^4+^ 47 in oxide glasses. These species have the same electron configuration as In^+^ centres and notably, the valence of these cations is mainly metastable. Thus, the number of powdered crystals containing these species is limited. One of our research interests is the local symmetry of these *n*s^2^-type centres in amorphous oxide glasses. Although emissions of In^+^ in alkali halides is conventionally recognised to have *O*_h_ symmetry^10^, it is expected that an In^+^ cation with a lone pair of electrons will have low symmetry in glasses with a random network. In addition, the origin of high quantum yield (QY) attained only in Sn^2+^-doped glass^39^,^40^ has not yet been clarified. Therefore, the emissions properties of an In^+^ emissions centre of the same electron configuration as Sn^2+^ in phosphate glass should be examined.

In the present study, we examined the PL properties of zinc phosphate glasses containing In^+^ emissions centres. Comparing the PL spectra and photodynamics with Sn^2+^-containing glasses exhibiting the same electron configuration, we determined the PL properties characteristic of each element. We also examined the local coordination state of the In^+^ emissions centre in a random matrix in the context of the site symmetry of the metastable species. Based on the results of In X-ray absorption fine structure (XAFS) analysis, we determined the local coordination state of In in the glass.

## Results

### PL-PL excitation (PLE) spectra of In-doped zinc phosphate glasses

**Figure 1:**
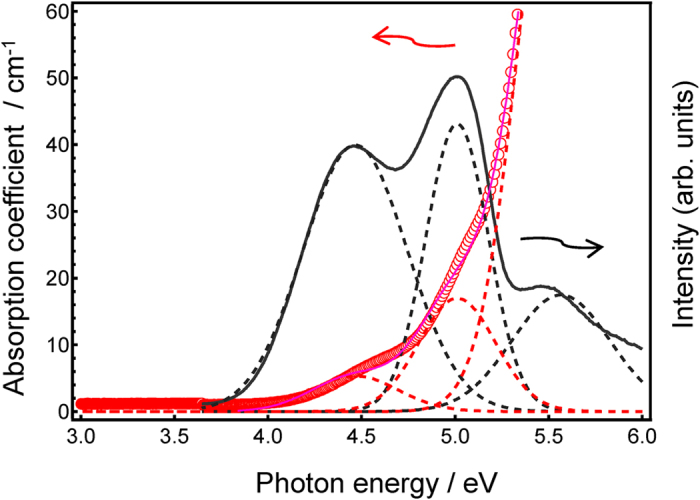
PLE spectrum of 1In_2_O_α_–60ZnO–40P_2_O_5_ glass along with the absorption spectrum. Dashed lines indicate three Gaussian functions after spectrum deconvolution. Each absorption band is attributable to a PLE band.

The transparent colourless In-doped 60ZnO–40P_2_O_5_ glasses have glass transition temperatures (*T*_g_s) of about 420 °C. Because the actual oxidation state of In has not been determined, the chemical composition is denoted as *x*In_2_O_α_–60ZnO–40P_2_O_5_. Figure 1 shows the PLE spectrum of 1In_2_O_α_–60ZnO–40P_2_O_5_ glass along with its absorption spectrum. Because Sn-free glass has an absorption edge at much higher photon energy (>6 eV) than the In-doped glasses, the observed absorption edge is attributed to the In species. Comparing the absorption spectrum with the PLE spectrum, both spectra had at least three excitation bands. After peak deconvolution, the absorption spectrum could be constructed using three Gaussian peaks with peak energies corresponding to those of the PLE bands. In a KI:In^+^ crystal, there are three excitation bands: A (~4.5 eV), B (~5.0 eV), and C (~5.6 eV)^17^. Because emissions are difficult to observe in 60ZnO–40P_2_O_5_ glass^39^,^40^, it is likely that the emissions in the In–doped 60ZnO–40P_2_O_5_ glasses originated from In^+^ cations. Although the starting material for the In species was trivalent In_2_O_3_, it has been reported that cations in a melt of phosphate glass prepared with ammonium phosphate tend to be reduced^43^.

**Figure 2:**
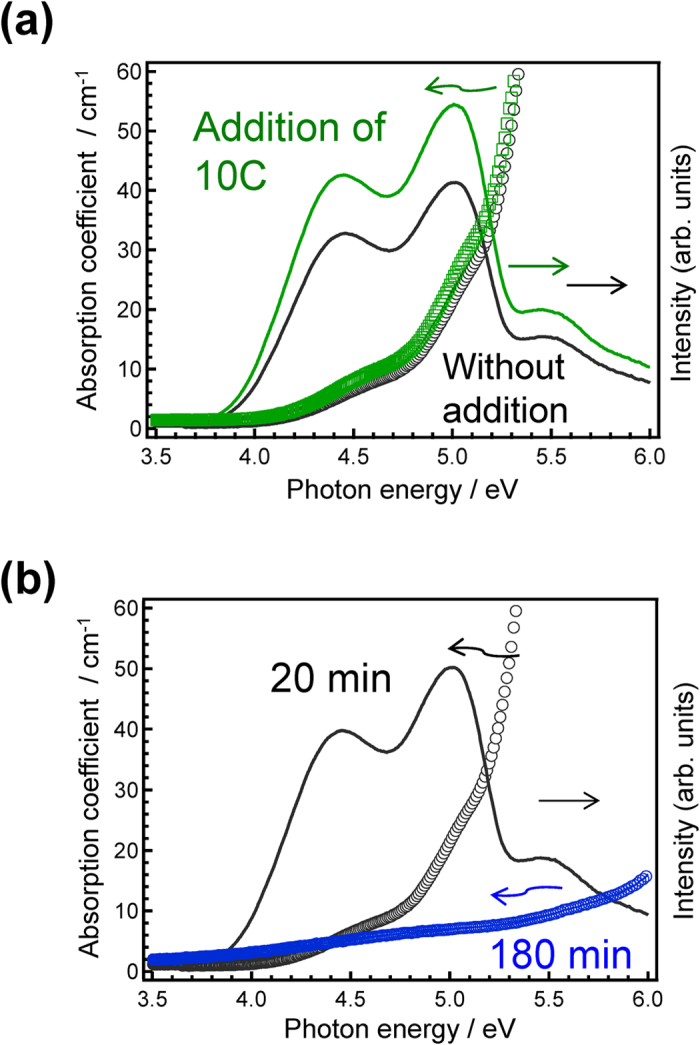
Correlation between the absorption and PLE spectra of In-doped zinc phosphate glasses prepared under different conditions. (**a**) PLE and optical absorption spectra of 1In_2_O_α_–60ZnO–40P_2_O_5_ glass with and without addition of 10 mol% C. (**b**) PLE and optical absorption spectra of 1In_2_O_α_–60ZnO–40P_2_O_5_ glass melted in air for 20 min and 180 min. The PLE spectra (solid lines) are normalised. The absorption spectra (dashed lines) indicate that absorption bands originating from In^+^ are correlated with emissions.

The redox state of the melt condition likely affects the In^+^ species generated from In_2_O_3_. The emissions intensity of 1In_2_O_α_–60ZnO–40P_2_O_5_ glass with addition of a reducing agent (10 mol% carbon) was 1.2 times that of non-doped 1In_2_O_α_–60ZnO–40P_2_O_5_ glass (Fig. 2a). Since the obtained carbon-added glass was transparent without residual carbon, we can conclude that the carbon, which worked as a reducing agent of In species, was burned off during the melting in the air. On the other hand, both the optical absorption edge and the PLE bands attributed to In^+^ species disappeared after melting in air for 3 h (Fig. 2b). Because an oxidation reaction of a metastable cation species during air-melting has been reported for another *n*s^2^-type cation^44^, metastable In^+^ may also be a transient species affected by melting conditions. Based on these redox reactions, we conclude that the three PLE bands in Fig. 1 are associated with different excitation processes of the In^+^ centre.

**Figure 3:**
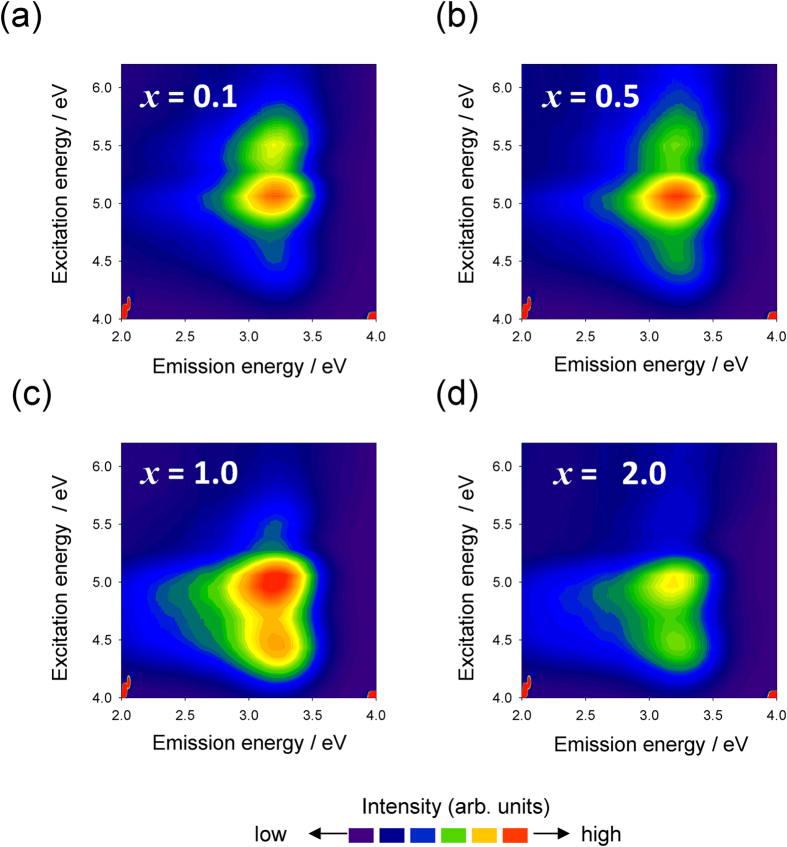
PL-PLE contour plots of *x*In_2_O_α_–60ZnO–40P_2_O_5_ glasses using an intensity axis on a linear scale. (**a**) *x* = 0.1, (b) *x* = 0.5, (c) *x* = 1.0, and (d) *x* = 2.0.

Figure 3 shows PL-PLE contour plots of *x*In_2_O_α_–60ZnO–40P_2_O_5_ glasses. For all glasses, we observed non-symmetric emission bands with long tails toward the lower photon energy region. All excitation bands (A, B, and C) exhibited emissions at 3.2 eV (Supplemental Fig. 1), which suggests that PL is independent of the excitation energy and that the energy level for radiative relaxation is fixed in these glasses.

**Figure 4:**
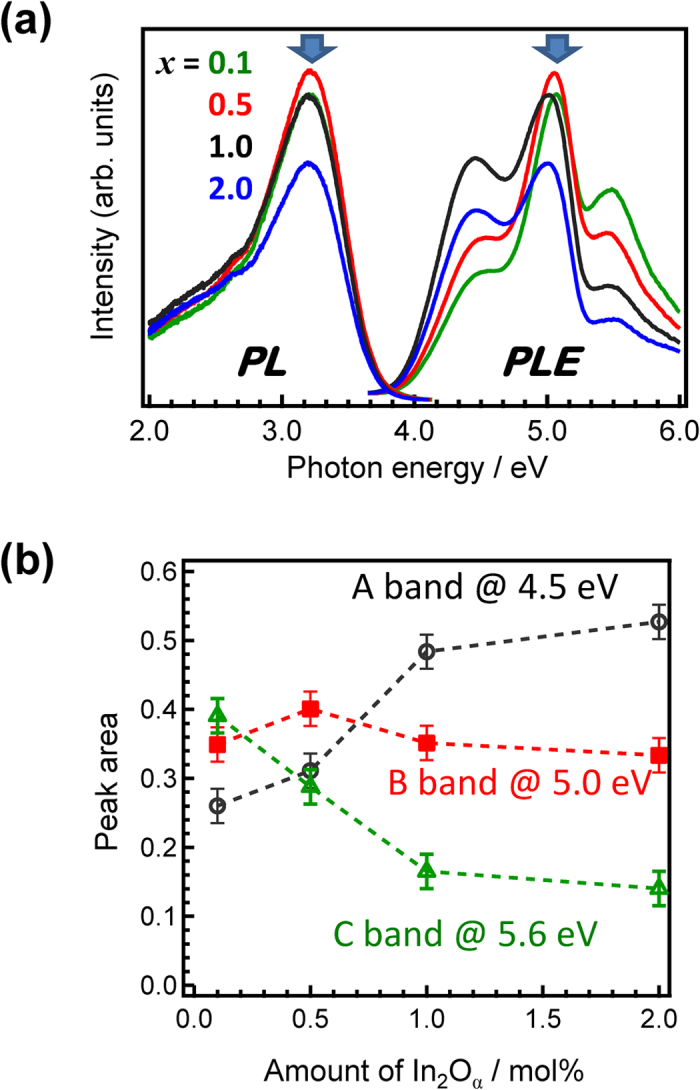
Concentration dependence of the PLE spectra of *x*In_2_O_α_–60ZnO–40P_2_O_5_ glasses. (**a**) PL-PLE spectra of *x*In_2_O_α_–60ZnO–40P_2_O_5_ glasses (*x* = 0.1, 0.5, 1.0, and 2.0). (**b**) Correlations between the amount of In_2_O_3_ and the peak ratios of the A, B, and C bands in the glass.

PL spectra were obtained by excitation of the B band (at ~5.0 eV), which gave the highest PLE peak intensities (Fig. 4). Broad emissions were observed with Stokes shifts of about 1.3 eV (Fig. 4a). The non-symmetric PL spectra suggested broad site distributions of the In^+^ centres in the glass. Considering the overlap of the three bands (Fig. 1), we can expect the obtained PL spectra to be affected by emissions from the other two bands. Correlations were observed between the ratio of each peak area and the amount of In_2_O_3_ (Fig. 4b). With increasing amounts of In_2_O_α_, the A band at 4.5 eV monotonically increased, whereas the C band at 5.6 eV decreased. For the Sn^2+^-doped glasses, two excitation bands were observed and the intensity of the lower excitation band increased with increasing amounts of Sn^2+^. Although the spectral shapes differ, the origin of the lower band for *n*s^2^-type centres should be essentially similar.

The QY of these glasses reached a maximum at ~0.5–1.0 mol% In. Although the Sn^2+^ centre, which has the same electron configuration, has high reported QY values of >80%^39^,^40^,^41^, the QY of the present In^+^-doped glass was about 20% with an excitation of 250 nm (4.96 eV, B band). It may be that a non-radiative path preventing effective photon conversion of In^+^ easily occurs from the excited state. On the other hand, a direct excitation band of the triplet state was observed in the In^+^-doped system. If ideal excitation and emission of an *n*s^2^-type centre occurred between the ground state and the triplet excited state, the QY of the radiative relaxation would be nearly 100%. Direct excitation to the triplet state is therefore attractive from the standpoint of effective energy conversion without singlet-triplet intersystem crossing.

### Photoluminescence dynamics of In-doped zinc phosphate glasses

**Figure 5:**
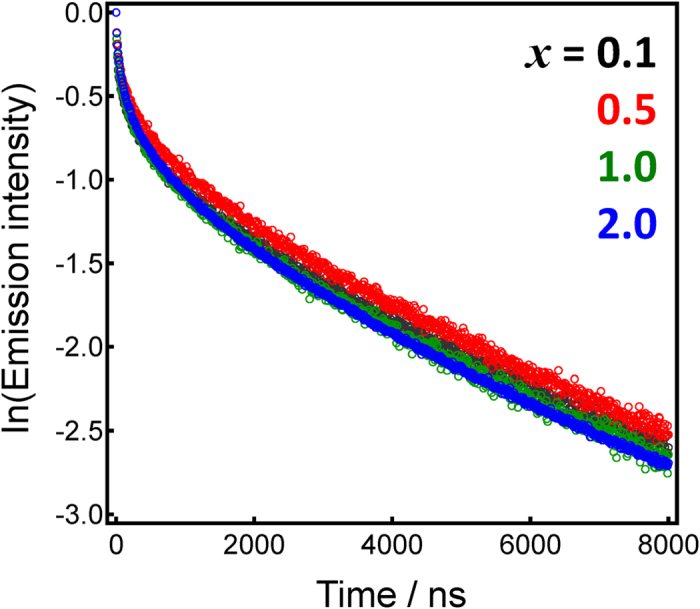
Intensity of emissions decay curves for *x*In_2_O_α_–60ZnO–40P_2_O_5_ glasses. Excitation and emissions energies were 4.43 eV and 3.14 eV, respectively.

**Figure 6:**
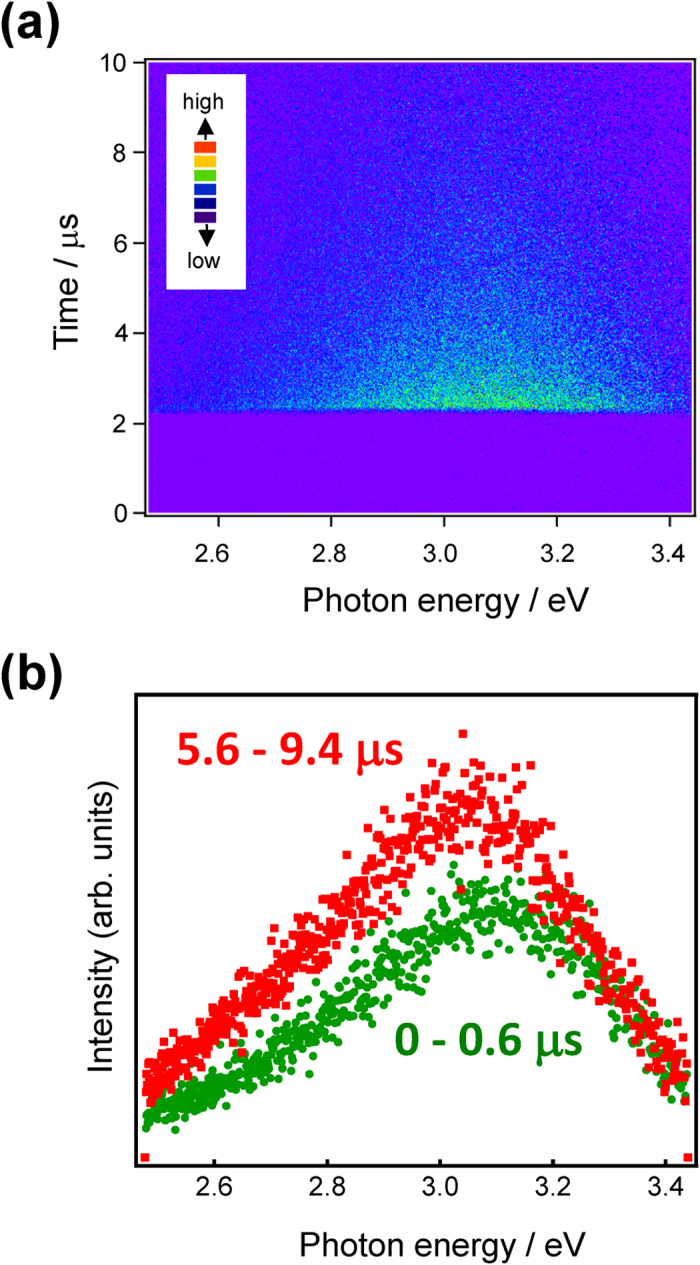
Time-dependent emissions properties of In_2_O_α_–60ZnO–40P_2_O_5_ glass. (**a**) Streak image of 1In_2_O_α_–60ZnO–40P_2_O_5_ glass irradiated at 250 nm. (**b**) Emissions spectra during different time periods calculated from the integral of the photon number.

Figure 5 shows emissions decay curves for the *x*In_2_O_α_–60ZnO–40P_2_O_5_ glasses. Non-exponential decay was slightly faster with increasing In concentration, consisting of at least two components: faster decay with a decay constant of sub-microseconds and slower decay with a decay constant of ~4 μs, suggesting triplet-singlet relaxation. To further examine emissions decay, a streak image of the glass was measured (Fig. 6a). The streak image indicated that 1) emissions decay was on a microsecond scale, classified as forbidden relaxation, and 2) non-exponential decay occurs with a peak shift toward lower photon energies. The decay constant τ_1/e_ of the In^+^ centre estimated from the streak image was about 4 μs, indicating that the final energy level was fixed independent of the excitation energy. Figure 6b shows the emission spectra at different times, calculated from the integral of the photon number. The initial peak emissions spectra were found to show red shift with time. This emissions property has also been observed in other *n*s^2^ centre-doped oxide glass phosphors^45^. Therefore, the obtained In^+^-doped glass phosphor may exhibit emissions properties similar to that of conventional emissions centres, other than the energy diagram.

### In K-edge XAFS measurement of In-doped zinc phosphate glasses

**Figure 7:**
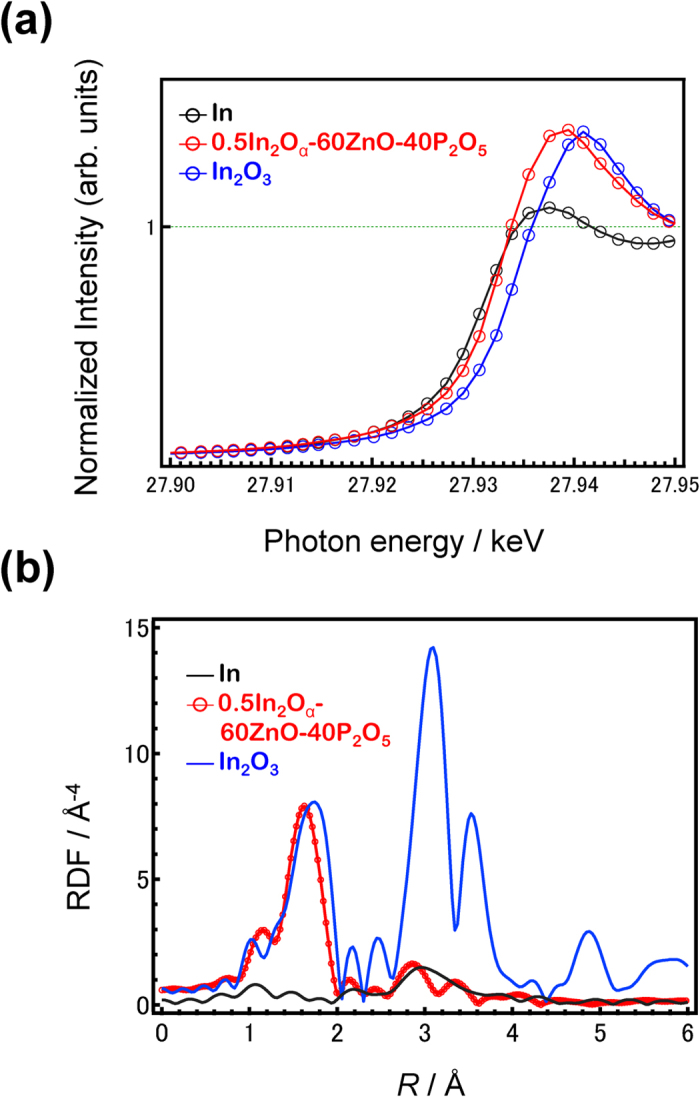
In K-edge XAFS analysis of In_2_O_α_–60ZnO–40P_2_O_5_ glass. (**a**) In K-edge XANES spectra of 0.5In_2_O_α_–60ZnO–40P_2_O_5_ glass along with In foil and In_2_O_3_. (**b**) FT of EXAFS spectra of the 0.5In_2_O_α_–60ZnO–40P_2_O_5_ glass along with In foil and In_2_O_3_.

Although the PL spectra and emissions dynamics suggest the existence of In^+^ species as *n*s^2^-type emissions centres, they do not identify the actual valence state of In. Therefore, the valence state of the In species was estimated using In K-edge X-ray absorption near edge structure (XANES) spectra (Fig. 7a). The shape of the spectrum of the In-doped glass was similar to that of In_2_O_3_, while the absorption edge was similar to that of In foil. Because a higher absorption edge indicates a higher oxidation state of the cation, we defined the absorption edge energy *E*_0_ as the energy at the zero-crossing of the second derivative. The In K-edge energy of the phosphate glass was higher than that of In, but lower than that of In_2_O_3_. |Δ(*E*_0_(In foil) – *E*_0_(glass, Ar))| and |Δ(*E*_0_(In_2_O_3_) – *E*_0_(glass, Ar))| were calculated to be 2.52 eV and 0.71 eV, respectively. Assuming the Δ*E*_0_ shift is proportional to the valence of the In species, the valence of In (α value) in the glass was ~2.5, suggesting that 25% of the In^3+^ was reduced to In^+^.

Figure 7b shows the Fourier transform (FT) of the extended XAFS (EXAFS) spectra of the glasses, In foil, and In_2_O_3_. Only the peak for the first coordination sphere of In was identified in the glasses, in contrast to the spectra for the standard materials in which the second coordination sphere was observed. The coordination number and the coordination distance of In in the glasses were estimated by fitting the first coordination sphere in the EXAFS spectra using the back-scattering factor and the phase shift extracted by fitting the In_2_O_3_ standard^49^ (Table 1). The results indicate that In in the glasses had a shorter In-O bond length and a smaller coordination number (<6, the coordination number of In_2_O_3_). From the In K-edge XANES spectra, we conclude that some amount of In exhibited a lower valence state in the glass. Therefore, the calculated parameters reflect the average coordination state of the In species, In^3+^ and In^+^, in the glass.

## Discussion

Our results demonstrate the luminescent properties of In^+^ centres and their local coordination state in oxide glass. Although In^+^ has the same electron configuration as other 5s^2^-type emissions centres such as Sn^2+^ 39,40,41,42,43,44,45, Sb^3+^ 46, and Te^4+^ 47, clear splitting of the PLE bands has not been observed in other systems. After peak deconvolution, the absorption and PLE bands were attributed to three excitation bands: A (~4.5 eV), B (~5.0 eV), and C (~5.6 eV) (Fig. 1). Bands A and B are associated with spin-forbidden transitions (^1^*S*_0_ → ^3^*P*_1_, ^1^*S*_0_ → ^3^*P*_2_), whereas B and C is attributed to a spin-allowed transition (^1^*S*_0_ → ^1^*P*_1_). This assignment is consistent with previous reports on In^+^ emissions bands in alkali halides^10^,^17^,^18^,^19^,^20^,^21^,^22^,^23^, except for the relative PLE band intensity. If this assignment is correct, it indicates that energy loss from phonon vibrations strongly affects intersystem crossing and decreases emissions intensity compared to the low-absorption region. Meanwhile, direct excitation of the singlet-triplet results in effective radiative relaxation, although the absorption intensity is much lower than that of conventional singlet-singlet allowed transitions. This assignment does contradict two conventionally accepted assumptions: 1) allowed transitions to a singlet excitation state have the highest PL intensities, and 2) conventional singlet or triplet states with *O*_h_ symmetry of the emissions centre are generally adaptable to real coordination. We hypothesize that non-uniform distribution of *n*s^2^-type emissions centres in glasses results in low-symmetry coordination. Thus, the local coordination state of emissions centres in oxide glasses is not necessarily a simple state exhibiting specific site symmetry.

The emissions dynamics indicate that In^+^ exhibits the luminescence properties of *n*s^2^-type emissions centres, although its emissions are not as high. The lower QY than that of Sn-containing glasses^39^,^40^,^41^ suggests that most of the In^3+^ was retained and not reduced to the metastable In^+^ species. Because In^+^ is a very sensitive and metastable species at higher temperatures, it may be quite difficult to attain 100% In^+^-doped oxide glass through conventional methods. Even for C-doped glass, the shape of the PLE spectrum was nearly the same as for the non-doped sample (Supplemental Fig. 2), indicating that the In^+^ concentration was not greatly increased. The similarity of the XANES and EXAFS spectra between the In-doped glasses and In_2_O_3_ also indicated that most of the In is present as In^3+^. Although determining the actual valence is difficult because of the lack of In_2_O, we estimate that the percentage of In^+^ among the total In in the glass was at most 25%.

Considering the local structure of a Sn^2+^ centre, which has a two^37^− or four-coordinated^44^ state in oxide glass, an In^+^ centre cannot exist without stabilization by a multi-coordination state with the lone pair of bridging oxygens in a glass network. As shown in Table 1 and Supplemental Fig. 3, the average coordination number of the In species was lowered, which indicates distorted coordination due to the lone pair on the In atom. Such unusual coordination is likely one cause of the large energy loss during intersystem crossing, or a higher transition probability to the triplet excitation state compared to symmetrical units.

In summary, we have demonstrated UV-induced PL in In^+^-doped phosphate glasses. A portion of the In^3+^ was reduced to In^+^, resulting in luminescence characteristic of *n*s^2^-type emissions centres with triplet-singlet relaxation. However, the excitation spectra consisted of three bands. Of these, the band with energy nearly equal to that of a spin-forbidden transition (^1^*P*_0_ -^3^*P*_1_) in alkali halides had the highest emissions intensity. Such an energy state is characteristic of random oxide networks and has not been previously reported. Demonstration of direct excitation to the triplet state will allow design of *n*s^2^-type doped oxide glasses with high quantum efficiencies. Meanwhile, the obtained results suggest that both singlet-singlet and singlet-triplet excitation bands in oxide glass may be tuneable by tailoring the local coordination field. The capacity for a disordered local structure is one of the merits of amorphous materials. Although the local coordination state of In^+^ in the glass is more disordered than that of In^3+^, the metastable glass network allows the existence of In^+^ centres even after annealing at the *T*_g_. Therefore, this demonstration of In^+^ species in glasses will be valuable for design of other In-containing materials.

## Methods

### Preparation of In-doped zinc phosphate glass

The In_2_O_α_–60ZnO–40P_2_O_5_ glasses were prepared by a conventional melt-quenching method using a platinum crucible^36^. The chemical composition of the glass was fixed at *x*In_2_O_α_–60ZnO–40P_2_O_5_ (in mol%, *x* = 0–2). As described previously^44^, batches consisting of ZnO and (NH_4_)_2_HPO_4_ were initially calcined at 800 °C for 3 h under ambient atmosphere. The calcined solid was mixed with In_2_O_3_ at room temperature and then melted at 1100 °C for 20 min under ambient atmosphere. The glass melt was quenched on a steel plate, held at 200 °C, and then annealed at the glass transition temperature *T*_g_ for 1 h. After cutting (10 mm × 10 mm × 1 mm), the glass samples were optically polished with aqueous diamond slurry.

### Analytical methods

The *T*_g_ was determined by differential thermal analysis at a heating rate of 10 °C/min using a TG8120 (Rigaku). The PL and PLE spectra were measured at room temperature using an F9000 fluorescence spectrophotometer (Hitachi). The absorption spectra were measured at room temperature using a U3500 spectrophotometer (Hitachi). The emissions decay at room temperature was measured using a Quantaurus-Tau (Hamamatsu Photonics) whose excitation light source was a 4.43-eV (280-nm) LED operated at a frequency of 10 kHz. The photoluminescence dynamics were also evaluated using a streak camera and a monochromator. The light source used for photoexcitation was an optical parametric amplifier system based on a regenerative amplified mode-locked Ti:sapphire laser (Spectra Physics) with a pulse-duration of 150 fs and a repetition rate of 1 kHz. The absolute QY of the glass was measured using a Quantaurus-QY (Hamamatsu Photonics).

### In K-edge XAFS measurement of In-doped zinc phosphate glasses

XAFS measurements were conducted at the In K-edge (27.9 keV) at the beam line BL01B1 at SPring-8 (Hyogo, Japan). The storage ring energy was operated at 8 GeV with a typical current of 100 mA. The measurements were carried out using a Si (311) double-crystal monochromator in the transmission mode (Quick Scan method) at room temperature. XAFS data for In foil and In_2_O_3_ were also collected under the same conditions. Curve fitting of the XAFS spectra was performed to determine the distances and coordination numbers using REX2000 software^50^. Values for the Debye-Waller factor and the phase shift of the glasses were obtained from fitted data for In_2_O_3_.

## Additional Information

**How to cite this article**: Masai, H. *et al.* Photoluminescence of monovalent indium centres in phosphate glass. *Sci. Rep.* **5**, 13646; doi: 10.1038/srep13646 (2015).

## Supplementary Material

Supplementary Information

## Figures and Tables

**Table 1:** First-shell In-O fitting results for *x*In_2_O_α_-60ZnO–40P_2_O_5_ glasses.

*x*	Coordination number	In-O distance (Å)
0.1	5.5	2.2
0.5	5.5	2.2
1	5.3	2.2
2	5.2	2.2
In_2_O_3_^49^	6.0	2.26

Reported results for In_2_O_3_^49^ are also shown for comparison.
