# Supplementary material for: Photoluminescence of monovalent indium centres in phosphate glass
Source: Sci Rep. 2015 Sep 1;5:13646. doi: 10.1038/srep13646 (PMC4555169; doi:10.1038/srep13646)
Supplement: Supplementary Information [file srep13646-s1.pdf]

Photoluminescence of monovalent indium centres in phosphate glass

H. Masai, Y. Yamada, S. Okumura, T. Yanaguida, Y. Fujimoto, Y. Kanemitsu & T. Ina

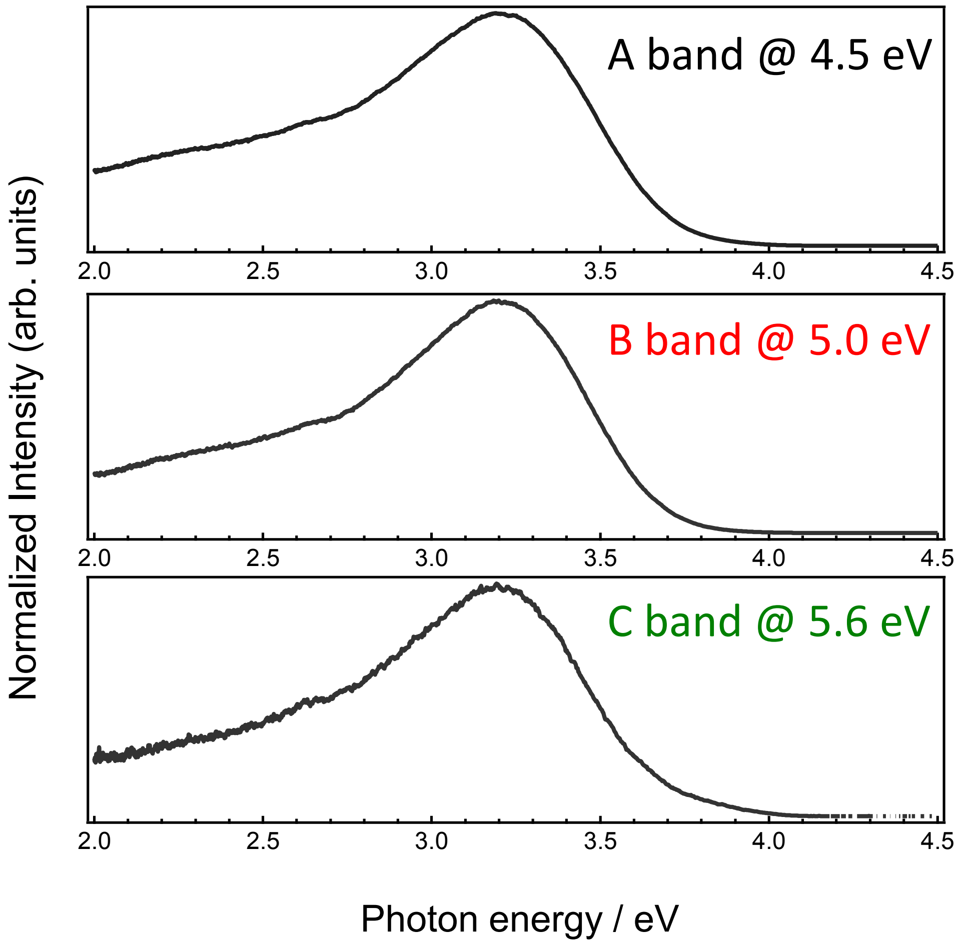

Supplemental Figure 1

PL spectra of $1In_2O_\alpha$-$60ZnO$-$40P_2O_5$ glasses obtained by excitation of different excitation bands. A (~ 4.5 eV), B (~ 5.0 eV), and C (~5.6 eV). All excitation bands (A, B, and C) exhibited emissions at 3.2 eV, which suggests that PL is independent of the excitation energy and that the energy level for radiative relaxation is fixed in these glasses.

Photoluminescence of monovalent indium centres in phosphate glass

H. Masai, Y. Yamada, S. Okumura, T. Yanaguida, Y. Fujimoto, Y. Kanemitsu & T. Ina

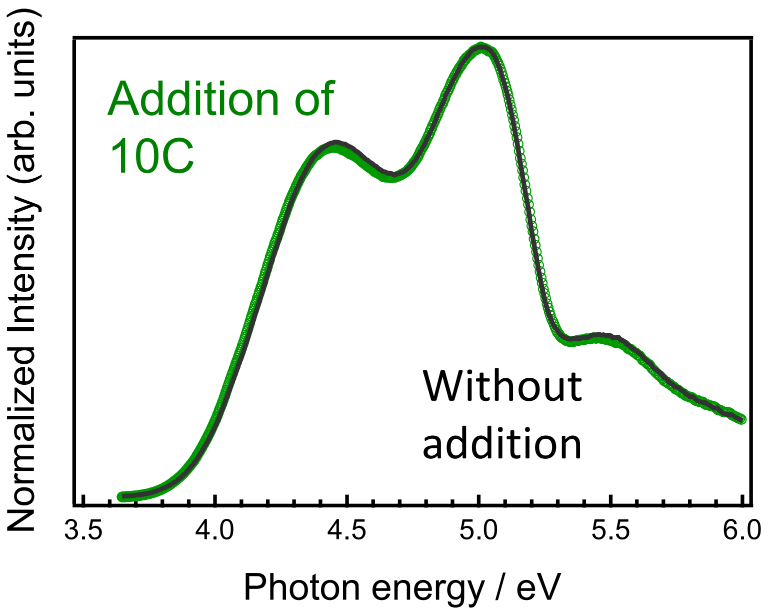

Supplemental Figure 2

Intensity-normalized PLE spectra of $1In_2O_3$-60ZnO-$40P_2O_5$ glasses with and without addition of 10 mol% of C.
The similarity of the two spectra indicates that the PLE shape mainly depends on the chemical composition (local coordination) of the glass. If residual carbon existed in the glass melt, we can see small carbon dots at the surface of the glass, because of the wettability between carbon and glass melt. Since we cannot observe the residual carbon using optical absorption or XRD measurement, we can conclude that that the carbon, which worked as a reducing agent of In species, was burned off during the melting in the air.

Photoluminescence of monovalent indium centres in phosphate glass

H. Masai, Y. Yamada, S. Okumura, T. Yanaguida, Y. Fujimoto, Y. Kanemitsu & T. Ina

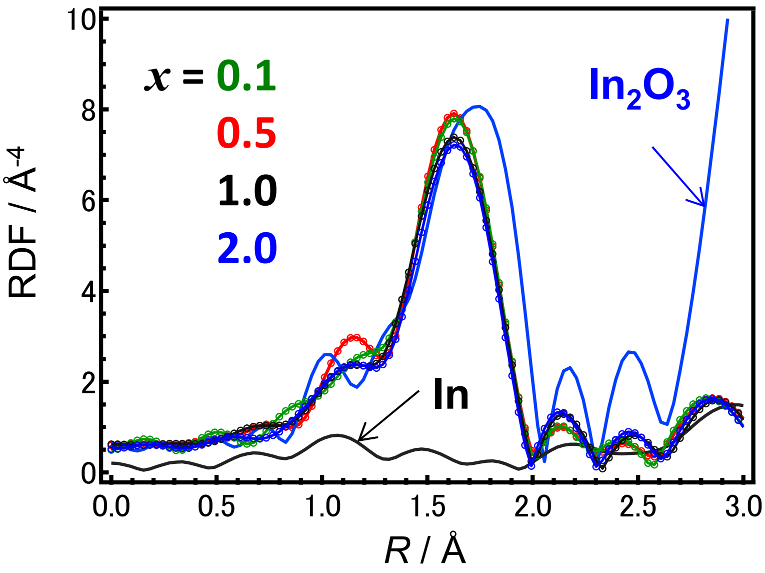

Supplemental Figure 3

FT of EXAFS spectra of the $x In_2O_\alpha$–60ZnO–40P_2O_5 glasses along with In foil and In_2O_3. A decrease in the RDF intensity suggests a decrease in the coordination number.
